# Hepatocellular Carcinoma With Right Atrium Metastases

**DOI:** 10.7759/cureus.23416

**Published:** 2022-03-23

**Authors:** Andreia M Teixeira, Iara Ferreira, Ana L Barbosa, Samuel Fonseca

**Affiliations:** 1 Internal Medicine, Centro Hospitalar Entre Douro e Vouga, Santa Maria da Feira, PRT

**Keywords:** cardiac tumours, cardiac metastases, cirrhosis, hepatocellular carcinoma, chronic liver disease

## Abstract

Hepatocellular carcinoma is common in patients with cirrhosis regardless of the etiology. Its presentation is uncommon in patients without known cirrhosis. It can spread commonly to the lungs, abdominal lymph nodes and bone but cardiac metastases are rare. The screening and early diagnosis impact the treatment feasibility and prognosis. The most common etiologies of cirrhosis and hepatocellular carcinoma are alcohol consumption and viral hepatitis B and C, however, non-alcoholic fatty liver disease (NAFLD) is becoming a more pronounced known risk factor for steatosis, advanced liver fibrosis, cirrhosis, and thus hepatocellular carcinoma due to the rise of metabolic syndrome prevalence. Although a known risk factor, there are no current recommendations for cancer surveillance in patients with NAFLD. The aim of this paper is to raise awareness of this rising complication by describing a rare initial presentation of hepatocellular carcinoma.

## Introduction

Hepatocellular carcinoma is a leading cause of cancer-related death and morbidity worldwide [1]. It occurs more often in males than females (2.4:1). Cirrhosis is the most important risk factor for the development of hepatocellular carcinoma despite the etiology [1]. Excessive alcohol consumption and viral B and C hepatitis are among the most prevalent etiologies, although non-alcoholic fatty liver disease (NAFLD) is becoming an emerging concern.

Among patients with chronic liver disease or cirrhosis, follow-up and screening for hepatocellular carcinoma must be pursued as early diagnosis can reduce morbimortality. Screening modalities, published in the European Association for the Study of the Liver (EASL) guidelines, encompass ultrasound (US) at a six-month interval as serological tests are not currently cost-effective [2]. The role of surveillance for patients with NAFLD without cirrhosis is not clear [2].

The diagnosis of hepatocellular carcinoma is made through ultrasound (US) in which nodules <1 cm that cannot be defined should be followed up with repeated US for 3-4 months and nodules >1 cm should undergo further investigation with either contrast-enhanced triple or quadriphasic computerized tomography or magnetic resonance [1]. In the former, an increased arterialization (wash-in phase) and decreased presence of contrast agents during the portal phase (washout) appear in hepatocellular carcinoma nodules [3], with no need for pathologic confirmation [1] in patients with cirrhosis.

Prognostic assessment should be performed including tumor burden, liver function and performance status [1, 2]. The most widely accepted staging system in clinical practice is the Barcelona Clinic Liver Cancer Staging System (BCLC) [4]. There are some treatment options for hepatocellular carcinoma, although liver resection and transplantation are treatment choices in early-stage tumors [1, 2].

## Case presentation

**Figure 1:**
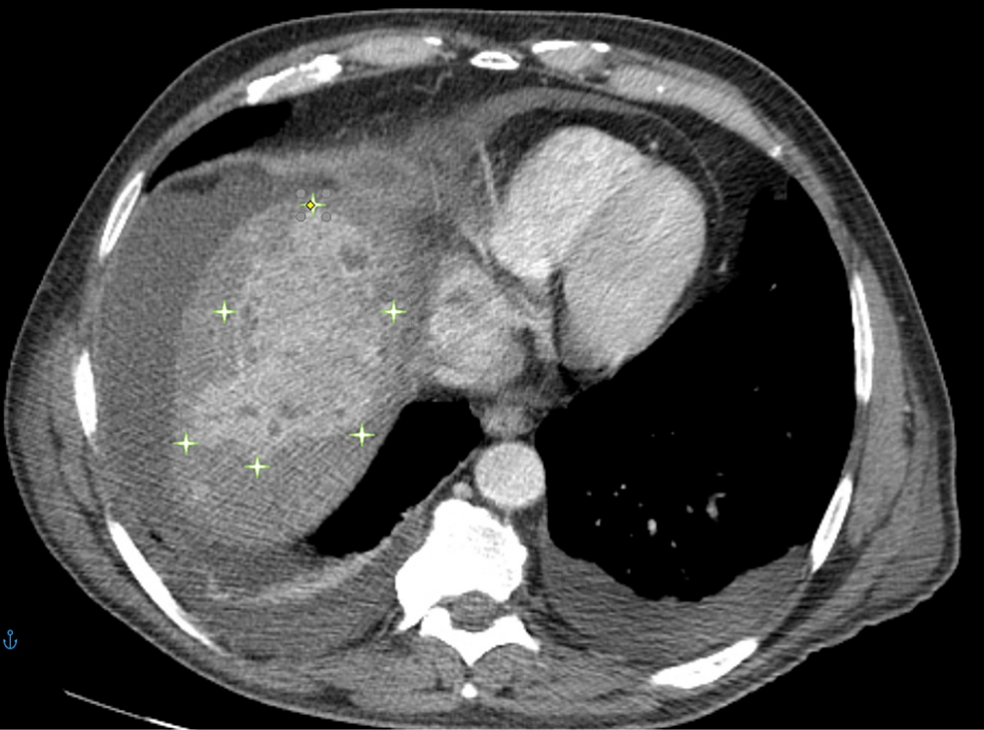
Computerized tomography (CT) with angiography Computerized tomography with angiography showing a liver mass compatible with hepatocarcinoma (outlined by the stars).

**Figure 2:**
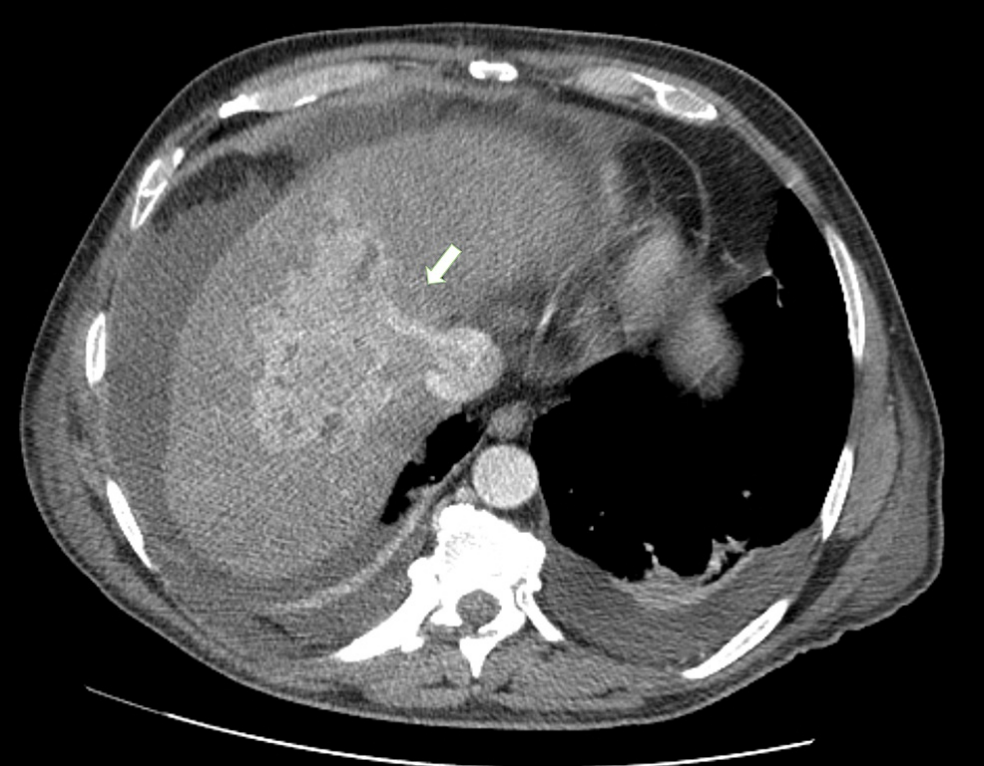
Computerized tomography (CT) with angiography with vena cava invasion Computerized tomography (CT) with angiography showing a liver mass compatible with hepatocellular carcinoma with vena cava invasion (arrow).

**Figure 3:**
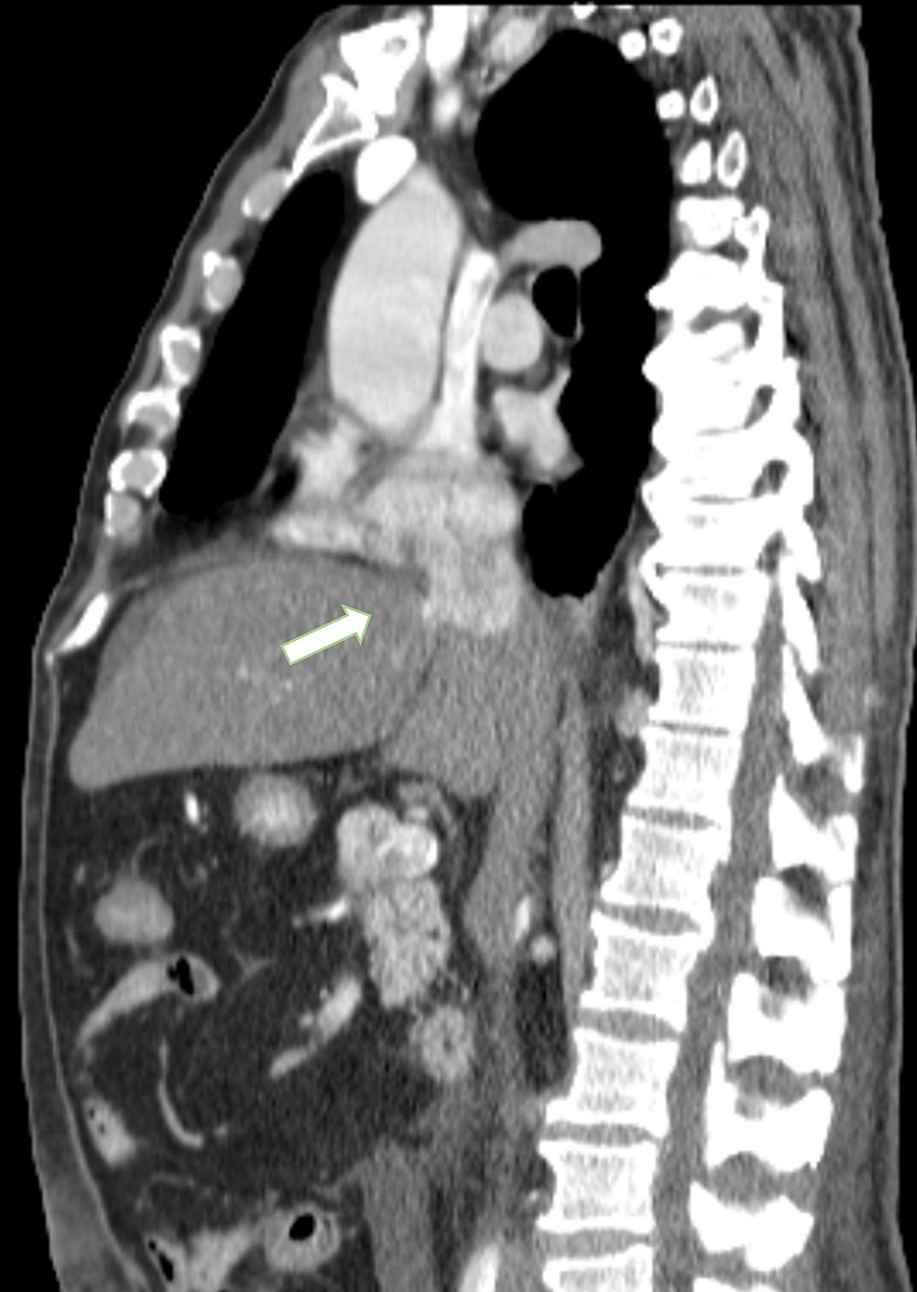
Computerized tomography (CT) with angiography with right atrium invasion Computerized tomography (CT) with angiography showing contiguous right atrium invasion (arrow).

A 66-year-old man, overweight, with diabetes mellitus for 15 years with poor metabolic control, bilateral retinopathy and known steatosis in an ultrasound performed in 2011, presented to the emergency department with exertional dyspnoea and peripheral oedema for 10 days. At admission, he was conscious and presented with a distended abdomen with a palpable mass in the right hypochondrium, ascites and peripheral oedema, respiratory insufficiency, low-normal blood pressure (100-106/70-75 mmHg), normal heart rate (75-85 beats per minute), regular pulse, and without heart murmurs. There was no jaundice, vascular spiders, and collateral circulation. Blood tests showed no alterations at haemogram, leucogram and platelet count, normal total bilirubin (0.85 mg/dL) with hepatocellular enzymes slightly greater than the normal references (aspartate aminotransferase 149 U/L; alanine aminotransferase 167 U/L, gamma-glutamyltransferase 261 U/L and lactate dehydrogenase 579 U/L). The patient denied excessive alcohol consumption, serologic testing for hepatitis B and C virus was negative and transferrin saturation was normal. A computerized tomography revealed signs of chronic liver disease with angiography revealing a hypervascular mass in the right lobe with 100x63 mm (Figure 1) compatible with hepatocarcinoma by the vascular characteristics in the different phases of contrast agents (Figure 2). It also revealed direct invasion of the inferior vena cava and right atrium (Figure 3). A transthoracic echocardiography showed an echogenic mass occupying the inferior vena cava and right atrium with little mobility along the cardiac cycle with borderline preserved right ventricle function (Tricuspid Annular Plane Systolic Excursion of 14 mm) and preserved left ventricle function.

Considering this data, the diagnosis of hepatocellular carcinoma with right atrium metastases was performed and a multidisciplinary discussion involving internal medicine, oncology, hepatobiliary surgery team and cardiology decided for symptomatic treatment due to tumor burden and performance status. No anticoagulation was initiated due to non-existent right-left communication and lack of literature evidence. The patient rapidly progressed with signs of right ventricular dysfunction and succumbed one week after hospitalization.

## Discussion

Most patients with hepatocellular carcinoma have underlying cirrhosis and it is uncommon to have a diagnosis of hepatocellular carcinoma without cirrhosis due to its natural evolution [5]. There are numerous risk factors for chronic liver disease and cirrhosis with excessive alcohol consumption, chronic B and C viral hepatitis the most prevalent [1]. Diabetes, non-fatty alcoholic liver disease, male gender, aflatoxins, smoking and metabolic and genetic disorders such as hemochromatosis and Wilson’s disease are known risk factors [1]. Our patient had diabetes for 15 years with poor metabolic control with organ damage, obesity, and had an ultrasound 10 years prior with steatosis. Although there are no straightforward recommendations for the screening and surveillance of liver disease for patients with NAFLD, it should be a concern since diabetes and obesity are becoming relevant causes of steatohepatitis, advanced fibrosis, and a growing indication for liver transplantation [1].

The most common organs for extrahepatic metastases are lungs, abdominal lymph nodes and bone, while cardiac metastases are rare [5]. The incidence of metastatic hepatocellular carcinoma to the right heart cavity reported to be less than 6% in autopsy series [5], generally due to contiguous extension via tumor thrombus to the inferior vena cava (such in our patient's case), lymphatic or hematologous spread [6]. The overall prognosis in cases of hepatocellular carcinoma with cardiac metastases is poor [7], due to the risk of sudden cardiac arrest [6], with no consensual treatment options. However, surgical and non-surgical interventions may play a palliative role to relieve lethal hemodynamic instability [7], with a multidisciplinary assessment team being of most importance for the decision of individualized treatment feasibility. Unfortunately, in this case, the patient progressed with rapid deterioration limiting further diagnostic and treatment techniques.

## Conclusions

Hepatocellular carcinoma has a major burden as it is one of the major causes of morbimortality worldwide. The recognition of risk factors for chronic liver disease and cirrhosis with adequate screening of hepatocellular carcinoma is of great importance as liver transplantation is the only curative treatment with the need for an early diagnosis. As cardiac metastases, due to the invasion of hepatocellular carcinoma, is a rare presentation of the disease with poor prognosis, few palliative treatment strategies, and no consensual recommendations being mandatory, an individualized decision is made by a multidisciplinary team after assessment of liver function, performance status and tumor burden.
